# A preterm infant with semilobar holoprosencephaly and hydrocephalus: a case report

**DOI:** 10.1186/1757-1626-3-35

**Published:** 2010-01-22

**Authors:** Ashish O Gupta, Patrick Leblanc, Krishna C Janumpally, Pattaraporn Tanya

**Affiliations:** 1 Department of Pediatrics, The Brooklyn Hospital Center, Brooklyn, New York 11201, USA

## Abstract

**Introduction:**

Holoprosencephaly (HPE) is a rare presentation in newborns. It refers to an incomplete or absent division of the prosencephalon or forebrain into distinct cerebral hemispheres.

**Case presentation:**

A preterm baby girl, first of dizygotic twins, born at 26 weeks of gestation to a 45 year old mother, was prenatally diagnosed with ventriculomegaly on fetal ultrasonogram. At birth the baby had frontal bossing with sun setting eyes and a full anterior fontanel. Initial head ultra-sonography (HUS) showed ventriculomegaly and semilobar holoprosencephaly, which was confirmed by computed tomography scan. Subsequently, the baby developed hydrocephalus that progressively increased. Eventually, the cerebrospinal fluid required drainage and a ventriculo-peritoneal shunt was placed.

**Conclusion:**

Holoprosencephaly has heterogeneous etiologies, including teratogenic and or a genetic basis. It is prudent to diagnose holoprosencephaly prenatally and determine the type to classify severity, complications and survival rate. It is also important to recognize that even with monozygotic twins only one twin may have HPE. The parents of a baby diagnosed with holoprosencephaly should be counseled about the poor prognosis.

## Introduction

Holoprosencephaly (HPE) represents an incomplete or absent division of the prosencephalon (forebrain) into distinct cerebral hemisphere usually occurring between 18^th ^and 28^th ^day of gestation [1,2]. HPE has an incidence rate of 1:250 inutero. However, the live birth rate is 1:16,000. This discrepancy is due to a high number of intrauterine deaths [2]. Holoprosencephaly is classified into 4 types depending on the degree of involvement of the forebrain and include: alobar, semilobar, lobar and a middle interhemispheric fusion variant. We describe a case of a preterm newborn diagnosed with holoprosencephaly and briefly discuss the pathogenesis, management and prognosis.

## Case presentation

A preterm dizygotic twin baby was born at 26 weeks of gestational age to a 45 year old mother (G1P0A0L0) by cesarean section. The APGAR scores at 1, 2 and 5 minutes were 6, 7 and 9 respectively. Respiratory effort remained poor. The baby was intubated with 2.5 mm endotracheal tube, was given positive pressure ventilation and was transferred to the Neonatal Intensive Care Unit (NICU).

In the NICU, the baby's weight was 765 grams. The head circumference was 24 cm and the length was 32.5 cm. Maturational assessment was performed using the Ballard Scoring method and was consistent with a gestational age of 26 weeks. The vital signs were within normal limits. Physical examination of the head exam revealed frontal bossing, a full anterior fontanel, wide open sutures and sun setting eyes. Neurologically, the baby had poor tone and reflexes.

The maternal history was unremarkable for any prenatal infections, trauma, drug abuse or any other chronic disease. No significant obstetric or family history was elicited. Prenatal ultrasound of the twins at 18 weeks gestation showed ventriculomegaly of the brain in one of the twin babies. Amniocentesis was performed and failed to show chromosomal abnormalities (46 XX).

**Figure 1:**
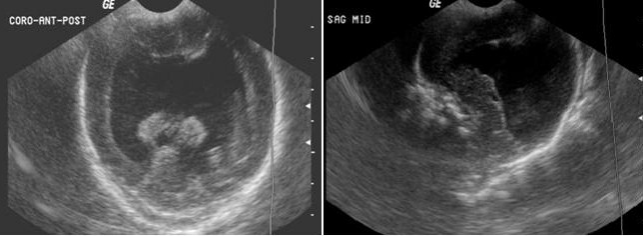
**Head ultra sonogram (USG) on day 2 of life showing single large monoventricle, fused thalami, hydrocephalus and thin brain mantle**.

On day 2 of birth, a head ultra sonogram (HUS) was performed due to a suspicion of increased intracranial pressure (full anterior fontanel, sun setting eyes, poor tone and reflexes) and to correlate with the prenatal ultrasound findings. The differential diagnoses were hydrocephalus, congenital anomaly of the brain, intracranial or intraparenchymal hemorrhage, tumor, anencephaly and holoprosencephaly. HUS revealed moderate to severe bilateral hydrocephalus. There was a large echogenic, fluid filled structure in the midline that could represent a hemorrhage or some other proteinaceous material. The brain mantle was well visualized. It appeared to be thin and lacked sulcal pattern (Figure 1). The final impression was a large interhemispheric bleed vs. semilobar holoprosencephaly. Neurosurgical and genetics consultations were requested.

The geneticist suggested performing a brain CT or MRI, cardiac ECHO, renal sonogram and blood work for microdeletions/rearrangements of chromosomes (CGH testing). The ECHO was performed and showed a grossly normal heart. Renal sonogram revealed a normal right kidney but the left kidney could not be found. A repeat renal sonogram one month later showed both structurally normal kidneys in appropriate positions. The CGH testing was also performed but results were not immediately available.

**Figure 2:**
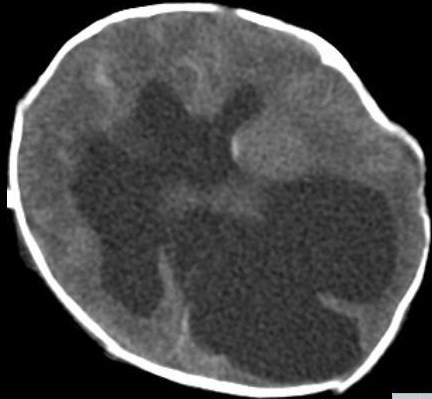
**CT scan of head, axial view; showing single large monoventricle with no sylvian fissure with extensive hydrocephalus and possibility of dorsal cyst**.

Neurosurgical consultation recommended a brain MRI or CT of the head following stabilization of the baby. Meanwhile HUS was repeated twice at 10 day intervals and showed similar findings as the previous study. At 2 weeks of age a CT scan of the head was performed and revealed semilobar holoprosencephaly with no sylvian fissure and a hypoplastic cerebellum (Figure 2). Neurosurgery recommended placement of ventriculo-peritoneal (VP) shunt once the baby reached a 2 kilogram weight. Ventricular tapping was recommended for increasing hydrocephalus or if the baby became symptomatic due to increased intracranial pressure (ICP).

The head circumference and clinical signs of increased intracranial pressures were monitored daily. The head circumference of the baby increased consistently each week (Table 1). At 5 weeks of age neurosurgery evaluated the baby for possible ventricular tap. On examination the anterior fontanel was full but not tense. There was no separation of metopic sutures. The CT scan findings and poor prognosis due the diagnosis of HPE were discussed with the parents. The mother declined a do not resuscitate (DNR) order.

**Table 1:** Weekly assessment of the baby for weight and head circumference.

Weeks	0	1	2	3	4	5	6	7	8	9	10	11	12
**GA**	26	27	28	29	30	31	32	33	34	35	36	37	38

**Wt (g)**	765	600	720	950	1085	1255	1344	1515	1935	2125	2183	2414	

**HC(cm)**	24	23	24	26.6	27.75	29.2	31.5	32	32.5	33.5	35	37	

**Proc**.							**T**	**T**	**T**				

GA- gestational age, Wt (g) - Weight (grams), HC-Head circumference, Proc- Procedure, T- CSF Taping done

At 6 weeks of age ventricular tapping of the cerebrospinal fluid was performed by neurosurgery. The baby tolerated the procedure. Subsequent examination and vital signs were within normal limits. Repeat CSF tappings were performed at the 7^th ^and 8th weeks of age. Neurosurgery reevaluated the baby at 10 week of age and recommended shunting (VP) when baby obtained a weight of 2.5 kilograms.

During the course of stay in the hospital, the baby developed respiratory distress, anemia of prematurity, neonatal jaundice, necrotizing enterocolitis, cholestasis, chemical rickets, presumed sepsis and electrolyte disturbances including hyponatremia, hypomagnesemia, hyperkalemia, and metabolic acidosis. Eye examination at 4 weeks of age showed stage1, zone 2 mild retinopathy of prematurity (ROP).

**Figure 3:**
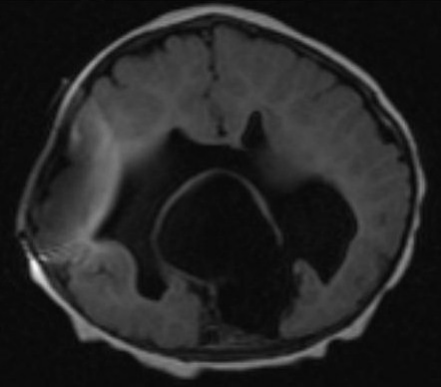
**MRI of brain after Ventriculoperitoneal shunt placement; Single monoventricle with Hydrocephalus, partially fused frontal interhemispheric fissure consistent with semilobar HPE**.

The baby was discharged home with home care. An appointment was made for a follow up evaluation with neurosurgery. A VP shunt was required and placed at 16 weeks of age. An MRI was done at 6 months of age and showed improved hydrocephalus with semilobar HPE (Figure 3). At 8 months of age the baby is under the 3^rd ^percentile on the growth chart. She is following her growth curve however, she is developmentally delayed. The other twin baby is growing well and achieving developmental milestones as is appropriate for the age.

## Discussion

Holoprosencephaly (HPE) or incomplete separation of forebrain, results from failure of induction and patterning of the rostral neural tube during early embryogenesis [3]. Multiple genetic and teratogenic factors have been implicated in patho-physiology of HPE. Multiple single gene disorders and chromosomal anomalies (55% cases) are associated with HPE. Mutation of the Shh gene is the most common cause of syndromic and familial HPE [3,4]. Trisomy 13 and Trisomy 18 are the most frequently identified chromosomal abnormalities accounting for 40% of the HPE cases [3,5]. Interestingly, no chromosomal abnormality was identified in our case. Also, the other twin child has had normal growth and development which further rules out any underlying genetic basis. In this case the twins were dizygotic and only one of them had HPE. However, even in monozogotic twins only one of babies may be affected with HPE [6]. Risk factors such as advanced maternal age (45 years), female gender, and prematurity (26 weeks) were present in this case and also concur with the normal pattern of HPE. Maternal obstetric and family history was otherwise unremarkable without any history of TORCH infections or drug abuse. The mother underwent amniocentesis during the pregnancy. Currently, no study has causally related amniocentesis to the development of HPE. Therefore, we could not determine the etiology of HPE in this case.

In most cases distinctive midline facial malformations are seen which correlate well with the degree of HPE and also have prognostic significance. The following facial features have been identified in HPE in descending order of severity- cyclopia (single midline fused eyes), ethmocephaly (ocular hypertelorism), cebocephaly (ocular hypertelorism with single nostril) and ocular hypertelorism with midline cleft [3,7]. The degree of clinical manifestation varies depending on the type of HPE. The most severe form is alobar HPE. Clinical manifestations include developmental delay, spasticity, seizure, hypo- or hypertonia, autonomic dysfunction, pituitary dysfunction, hypothyroidism, hypogonadism and most importantly feeding difficulty with increased risk of aspiration pneumonia and various other problems [2,7].

Diagnosis of HPE is usually made prenatally in high risk mothers by prenatal trans-abdominal or transvaginal ultrasound. Our case of HPE was diagnosed prenatally on ultrasound and later was classified as the semilobar type. MRI is considered the best modality to diagnose and classify HPE [3,4]. On MRI, alobar HPE results in a horseshoe shaped monoventricle and an absent hemispheric fissure. Semilobar HPE shows single ventricular cavity with partial separation and a partial interhemispheric fissure or falx (mostly posterior) [3,7]. Although MRI was not utilized in this case the diagnosis was clearly evident and MRI would not have changed the treatment and or prognosis.

In cases of HPE microcephaly is the most common presentation. Macrocephaly, if present is indicative of hydrocephalus in cases of HPE [3]. We observed hydrocephalus, an atypical feature in our patient, which was later on treated with ventriculo peritoneal (VP) shunt. Treatment of HPE, is usually supportive. VP shunt is used in the management of hydrocephalus. Fundoplication or gastrostomy tube may be necessary in cases of gastro esophageal reflux disease (GERD) and frequent aspiration pneumonia.

The prognosis of HPE is very poor and depends on the degree and type of HPE and the extent of facial dysmorphic features. Only 50% of patients with alobar HPE will survive by 4-5 months of age. Only 20% of these cases will survive by 12 months of age. Conversely, isolated semilobar and lobar types have a 50% survival rate beyond 12 months [3,8]. Recurrence of HPE in future pregnancies depends on the type of genetic mutation present and associated chromosomal abnormalities.

## Conclusion

HPE has heterogeneous etiologies that can include a teratogenic and/or a genetic basis. It is prudent to diagnose HPE prenatally and determine the type in order to classify the severity of HPE, the complications and rates of survival. MRI is the best modality for diagnosing and classifying the type of HPE. HPE babies require multidisciplinary treatment approach. The parents should be counseled about the poor prognosis and babies should be referred to early intervention for physical and occupational therapies.

## Consent

Written informed consent was obtained from the patient for publication of this case report and accompanying images. A copy of the written consent is available for review by the Editor-in-Chief of this journal.

## Conflict of interests

The authors declare that they have no competing interests.

## Authors' contributions

AOG took care of the patient, wrote first draft of the manuscript, and critically reviewed it. KCJ and PT gave valuable inputs to the draft; PL supervised treatment of the patient and reviewed the manuscript. All authors approved the final manuscript.
